# Infusion of allogeneic umbilical cord blood hematopoietic stem cells in patients with chemotherapy-related myelosuppression

**DOI:** 10.3892/etm.2014.2022

**Published:** 2014-10-15

**Authors:** YI YAO, QIBIN SONG, YUXIN CHU, HONGYUN GONG, NA LI, QINYONG HU, XIAOTAO XU

**Affiliations:** Cancer Center, Renmin Hospital of Wuhan University, Wuhan, Hubei 430060, P.R. China

**Keywords:** umbilical cord blood, hematopoietic stem cells, chemotherapy-related myelosuppression

## Abstract

Chemotherapy-induced myelosuppression is one of the main problems in the treatment of cancer. In the present study, the effects of allogeneic umbilical cord blood hematopoietic stem cell (UCB-HSC) infusion were investigated on the treatment of chemotherapy-related myelosuppression. In total, 65 patients (male, 42; female, 23) diagnosed with chemotherapy-related myelosuppression were included in the study. The majority of the patients were classified with stage II myelosupression at enrolment, and an average concentration of 7.07×10^9^/l UCB-HSCs were transfused through the peripheral vein. The minimum values of the white blood cell (WBC) count, hemoglobin (Hb) level, platelet (PLT) count and Karnofsky performance status (KPS) scores were recorded prior to and between days 7 and 14 following UCB-HSC infusion. When assessing the overall data, the results revealed that the mean WBC and PLT counts increased significantly following UCB-HSC infusion. However, the subgroup analyses based on gender and KPS score revealed that UCB-HSC infusion was more successful in male patients and those with a higher KPS score. Spearman’s correlation analysis revealed a linear correlation between the number of transfused UCB-HSCs and the changes in the WBC and PLT counts following treatment. In conclusion, the results indicated that peripheral vein infusion of non-human leukocyte antigen matched UCB-HSCs can markedly improve chemotherapy-related myelosuppression in a safe and effective manner.

## Introduction

Currently, conventional chemotherapy is one of the main approaches for the treatment of numerous forms of advanced cancer. Increasing the dose intensity (DI) of chemotherapeutic drugs is considered to enhance their ability to kill cancer cells and reduce the occurrence of drug resistance. However, increasing the DI is not feasible due to the resultant chemotherapy-induced myelosuppression (1,2). At present, strategies for the management of chemotherapy-induced myelosuppression are limited. Furthermore, the therapeutic effects vary significantly among individuals, and a number of adverse reactions have been observed. Therefore, an improved therapeutic method is urgently required to solve this problem.

In 1988, Dr Eliane Gluckman successfully treated a child with Fanconi anemia by performing the first umbilical cord blood (UCB) hematopoietic stem cell (HSC) transplantation, which demonstrated that UCB-HSC transplantation may be a promising tool for the treatment of blood system impairments (3,4). Traditional allogeneic UCB-HSC transplantation requires partial human leukocyte antigen (HLA) matching, ablation of the recipient’s marrow and immunosuppression following transplantation (5,6). These requirements significantly limit the broad application of UCB-HSC transplantation in patients with chemotherapy-related myelosuppression. However, a number of studies have shown that non-HLA matched allogeneic UCB-HSC transplantation exerts no risk of graft-versus-host disease (GVHD), and encouraging clinical therapeutic effects have been observed in the treatment of degenerative diseases (6–8). The underlying mechanism of the cord blood stem cells is hypothesized to be largely a result of the secretion of a number of growth factors, which are considered to be central in the repair of injured cells. However, transplanted UCB-HSCs are not permanently engrafted in the recipient’s bone marrow, as transplanted HSCs are eliminated by the host immune system after a certain period of time (6). These observations indicate that non-matched UCB-HSCs may be useful in the treatment of chemotherapy-related myelosuppression.

In the current study, the clinical observations following the application of allogeneic UCB-HSC transplantation for the treatment of chemotherapy-related myelosuppression were investigated.

## Materials and methods

### Subjects

In total, 65 patients (male, 42; female, 23; age range, 22–83 years; average age, 59.32 years) that had been diagnosed with chemotherapy-related myelosuppression were included in the study. Written informed consent was obtained from the patient. These patients had a variety of cancer types, including lymphoma, lung cancer, gastric cancer, colorectal cancer, nasopharyngeal carcinoma and breast carcinoma, all of which were confirmed by a biopsy examination. All the patients received the conventional dose of chemotherapy (1,2). The chemotherapy drugs administered were pemetrexed, gemcitabine, paclitaxel, docetaxel, cisplatin, carboplatin, nedaplatin, fluorouracil, oxaliplatin, irinotecan, cyclophosphamide, epirubicin and vindesine. Certain patients also received radiotherapy. The majority of patients enrolled were classified with stage II myelosuppression (as defined by National Cancer Institute common toxicity criteria) prior to the initiation of UCB-HSC infusion (1,2). A routine blood examination was conducted for each patient to determine the minimum values of the white blood cell (WBC) count, hemoglobin (Hb) level and platelet (PLT) count prior to the stem cell treatment, which were found to be 0.32×10^9^/l, 69 g/l and 25×10^9^/l, respectively. Drugs that may have affected the blood cell counts, including granulocyte colony-stimulating factor (G-CSF), granulocyte-macrophage colony-stimulating factor (GM-CSF), erythropoietin (EPO) and thrombopoietin (TPO), were avoided for at least two weeks prior to and two weeks following UCB-HSC infusion.

### Treatment procedure

UCB was donated by the Obstetrics Department of Renmin Hospital of Wuhan University (Wuhan, China), and was harvested in the third stage of labor. HSCs were prepared in the stem cell laboratory of the Cancer Center of Renmin Hospital. The study was approved by the Ethics Committee of Renmin Hospital. The procedures for UCB collection, separation, preservation and testing were performed according to a previously described protocol (8,9). UCB-HSCs that were confirmed to be non-HLA matched and non-ABO matched were stored in 100-ml suspensions, with an average concentration of 7.07×10^9^/l and cell viability of >90%. The cells were transfused via a needle with a diameter of 0.9 mm through the peripheral vein. To avoid a transfusion reaction and GVHD, 5 mg dexamethasone was regularly administered via an intravenous drip prior to transplantation. The vital signs of the patients were closely monitored during the 30-min period of stem cell infusion.

### Evaluation

Karnofsky performance status (KPS) scores were determined prior to and following UCB-HSC transplantation (10). In addition, routine blood tests were performed prior to treatment and between days 7 and 14 following treatment. Laboratory parameters, including the WBC and PLT counts and the Hb level, were recorded and analyzed.

### Statistical analysis

SPSS version 12.0 (SPSS, Inc. Chicago, IL, USA) for Windows was used for statistical analysis, and the data are expressed as the mean ± standard deviation. A paired t-test was used to analyze the differences between parameters prior to and following treatment. In addition, Spearman’s correlation analysis was performed to analyze correlations among the variables. All the calculated P-values were two-tailed, and P<0.05 was considered to indicate a statistically significant difference.

## Results

### Overall data analysis

As shown in Table I, the mean WBC count, Hb level and PLT count of the patients prior to treatment were 4.82±2.41×10^9^/l, 105.75±20.90 g/l and 154.20±94.51×10^9^/l, respectively. Following UCB-HSC transplantation, these values increased to 5.92±2.51×10^9^/l, 107.02±21.45 g/l and 172.66±105.08 × 10^9^/l, respectively, with the WBC count increasing by 22.98% (P=0.0001) and the PLT count increasing by 11.97% (P=0.01). These differences were found to be statistically significant.

### Subgroup analysis

When analyzed according to gender, the WBC count in the males (n=42) increased from 4.85±2.23×10^9^/l to 6.14±2.45×10^9^/l (P=0.003), while the PLT count increased from 150.88±96.50×10^9^/l to 181.62±112.84×10^9^/l (P=0.004). In the female group (n=23), a statistically significant difference was not observed in the PLT count following stem cell treatment. However, the WBC count increased significantly from 4.75±2.76×10^9^/l to 5.53±2.63×10^9^/l (P=0.03), which was a 16.44% increase (Table I).

Next, the patients were divided into two groups according to their KPS score (≥60 and <60 points). The WBC and PLT counts in the group with a KPS score of ≥60 (n=45) increased from 4.49±2.44×10^9^/l and 153.96±73.01×10^9^/l to 5.71±2.54×10^9^/l and 168.29±65.59×10^9^/l, respectively (P=0.0001 and 0.037, respectively). However, in the patients with a KPS score of <60 (n=20), no statistically significant differences were observed in the WBC count, PLT count or Hb level (Table I).

### Correlation analysis

**Figure 1:**
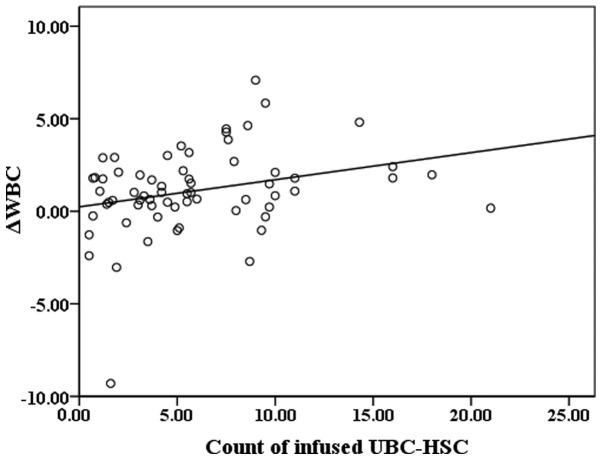
Linear correlation (*r*=0.289, P=0.019) between the number of transfused UCB-HSCs and the ΔWBC (WBC count after treatment - WBC count before treatment). WBC, white blood cell; UCB, umbilical cord blood; HSC, hematopoietic stem cell.

**Figure 2:**
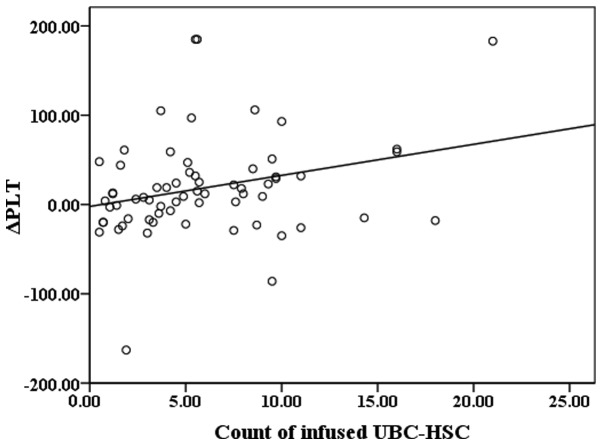
Linear correlation (*r*=0.271, P=0.029) between the number of transfused UCB-HSCs and the ΔPLT (PLT count after treatment - PLT count before treatment). PLT, platelet; UCB, umbilical cord blood; HSC, hematopoietic stem cell.

**Figure 3:**
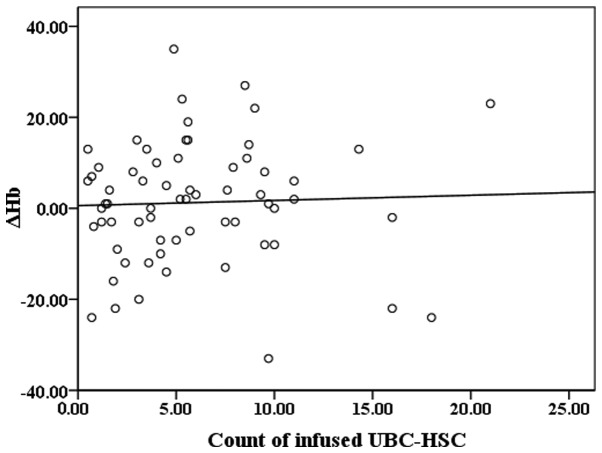
Linear correlation (*r*=0.095, P=0.449) between the number of transfused UCB-HSCs and the ΔHb (Hb level after treatment - Hb level before treatment). Hb, hemoglobin; UCB, umbilical cord blood; HSC, hematopoietic stem cell.

To determine whether the actual number of UCB-HSCs transfused into the patients was associated with the changes in the WBC count (Fig. 1), PLT count (Fig. 2) and Hb level (Fig. 3) following treatment, three scatter plots showing these data were constructed. Notably, the scatter plots revealed that the three parameters tended to change with the number of transfused stem cells. Spearman’s correlation analysis was subsequently conducted and linear correlations were observed between the number of transfused UCB-HSCs and the ΔWBC (WBC count following treatment - WBC count prior to treatment; correlation coefficient, 0.289; P=0.02) or the ΔPLT (PLT count following treatment - PLT count prior to treatment; correlation coefficient, 0.271; P=0.03).

### KPS score

Following UCB-HSC transplantation therapy, the majority of patients reported noticeable subjective improvements in numerous symptoms, including fatigue, anorexia, dizziness and chest congestion. As shown in Table I, the KPS scores increased significantly following stem cell treatment (P<0.01).

### Adverse reactions

No serious adverse reactions were observed in the stem cell treatment patients. In addition, there were no transfusion reactions, evidence of GVHD or cases of morbidity and mortality attributable to the stem cell transplant procedure.

## Discussion

In addition to surgery, chemotherapy is well-recognized as one of the most important anticancer interventions. The therapeutic efficacy and overall survival rate associated with chemotherapy are constantly increasing. However, chemotherapy-induced myelosuppression limits the possible intensity and progress of chemotherapy. Although several solutions to the problem of chemotherapy-induced myelosuppression have emerged in recent decades, including G-CSF, GM-CSF, interleukin (IL)-11, EPO, TPO or even blood component infusion (2), these therapies have been found to have certain disadvantages in clinical practice. For example, these therapeutic methods are accompanied by numerous adverse reactions, including edema, headache, fever, palpitation, nausea, vomiting, dizziness, insomnia, dyspnea, rash, conjunctival congestion and muscle and joint pain. Furthermore, their therapeutic effects vary greatly among individuals and the exact efficacy remains unknown (2,10,11).

Bone marrow is the source of all hematological cells in the human body, and the majority of blood cells are generated by HSCs or hematopoietic progenitor cells in the bone marrow. HSCs have the capacity to self-renew and generate multiple progenitor cell and mature cell types (12); thus, in theory, the cells are widely applicable in clinical practice. Compared with stem cells derived from the bone marrow and peripheral blood, those derived from the UCB may be a better source for transplantation due to their various advantages, including their rich source, strong regeneration ability and weak antigenicity to cord blood lymphocytes, as well as the low incidence and severity of GVHD with their use. Furthermore, UCB-HSC transplantation can be performed even if there are one or two differences in the HLA between the donor and recipient. Therefore, this treatment does not require any HLA matching or use of immunosuppressives (13). Moreover, the incidence of infection following UCB-HSC transplantation is low, the response is rapid and the cost is relatively low. Thus, UCB-HSC transplantation may be a promising therapy in numerous fields of medicine (13–15).

Previous studies have reported that the use of UCB-HSCs in the treatment of myelosuppression may activate the bone marrow (5,7,19). UCB has been shown to be abundant in hematopoietic growth factors, including G-CSF (16), GM-CSF (5), EPO (16), IL-2 (16), IL-6 (16,17), insulin-like growth factors (18) and steel factor (7). The expression levels of these growth factors are significantly higher in UCB compared with the peripheral blood. Following transfusion, UCB-HSCs strongly activate stem cells in the bone marrow, thereby playing an important role in the treatment of myelosuppression (5,7,19). Zhang *et al* (7) summarized the clinical function of UCB-HSCs as follows: i) Specific stimulation, where UCB is able to stimulate hematopoiesis in the host to reverse anemia and granulocytopenia; and ii) non-specific stimulation, where the subjective symptoms of the patients improve significantly following UCB transplantation.

UCB is rich in not only hematopoietic growth factors, but also HSCs, which may be responsible for producing the majority of the aforementioned growth factors (5). The HSCs in UCB release hematopoietic growth factors and stimulate hematopoiesis following infusion. Subsequently, the function of hematopoiesis is gradually restored in the activated bone marrow. An *et al* (20) treated 52 patients with chemotherapy-and radiotherapy-induced myelosuppression using UCB-HSC transplantation, and found that the WBC and red blood cell counts in the patients significantly and rapidly improved following treatment, although the increase in the PLT count was less significant.

In the present study, the 65 patients exhibited similar results, with only a few differences. Furthermore, GVHD did not occur in any patient. Following transplantation, the majority of the patients achieved a higher KPS score and marked improvements in a number of clinical symptoms, including fatigue, loss of appetite, dizziness and chest congestion. At two weeks after treatment, the blood circulation impairment caused by myelosuppression recovered, with the WBC and PLT counts increasing by an average of 22.82 and 11.97%, respectively. However, the difference in the Hb level prior to and following treatment was not significant. Additional subgroup analysis based on the gender and KPS scores revealed that male patients and those with a good KPS score appeared to benefit more from stem cell transplantation compared with female patients and those with a poor KPS score, respectively. In addition, the ΔWBC and ΔPLT in each patient was found to positively correlate with the number of stem cells transfused. These observations indicated that increasing the number of stem cells transfused may promote the restoration of bone marrow function in patients with myelosuppression. The possibility that the bone marrows of male patients and patients with a good performance status showed a better response to growth factors released by UCB-HSCs was considered. Furthermore, it was hypothesized that UCB-HSCs stimulate different types of blood cells via different mechanisms. In future studies, these issues should be addressed.

In conclusion, the results of the present study demonstrated that peripheral vein infusion of non-HLA matched UCB-HSCs markedly improved chemotherapy-related myelosuppression in a safe and effective manner. In addition, UCB-HSC infusion was shown to significantly ease the symptoms of myelosuppression; however, the therapy was more effective in male patients and patients with a good KPS status. In order to achieve greater therapeutic benefits, increasing the number of UCB-HSCs in each infusion, or the number of infusions, may be considered. Further research into the subgroup differences observed in the present study and potential strategies for increasing the number of stem cells delivered per infusion are currently under investigation.

## Figures and Tables

**Table I:** Values of three blood-related parameters and KPS scores prior to and following UCB-HSC transplantation.

Groups	WBC (×10^9^/l)	PLT (×10^9^/l)	Hb (g/l)	KPS
Before treatment				
Total	4.82±2.41	154.20±94.50	105.75±20.90	60.00±19.28
Males	4.85±2.23	150.88±96.50	109.52±20.00	59.28±19.68
Females	4.75±2.76	160.26±92.57	98.87±21.16	61.30±18.90
KPS ≥60	4.49±2.44	153.95±73.01	109.07±21.78	70.89±9.25
KPS <60	5.56±2.24	154.75±133.19	98.30±16.96	35.50±11.91
After treatment				
Total	5.92±2.51^b^	172.66±105.08^b^	107.02±21.45	67.23±18.83^b^
Males	6.14±2.45^b^	181.62±112.83^b^	108.90±22.73	65.00±18.38^b^
Females	5.53±2.62^a^	156.30±89.24	103.56±18.89	71.30±19.38^b^
KPS ≥60	5.71±2.54^b^	168.29±65.59^a^	110.00±20.25	77.33±8.89^b^
KPS <60	6.42±2.43	182.50±164.58	100.30±23.06	44.5±15.04^b^

Results are expressed as the mean ± standard deviation.

a P<0.05 and

b P<0.01, vs. values prior to treatment.

WBC, white blood cell; PLT, platelet; Hb, hemoglobin; KPS, Karnofsky performance status; UCB, umbilical cord blood; HSC, hematopoietic stem cell.

## References

[1] Yeshurun M, Labopin M, Blaise D (2014). Impact of postremission consolidation chemotherapy on outcome after reduced-intensity conditioning allogeneic stem cell transplantation for patients with acute myeloid leukemia in first complete remission: a report from the Acute Leukemia Working Party of the European Group for Blood and Marrow Transplantation. Cancer.

[2] Dale DC, McCarter GC, Crawford J, Lyman GH (2003). Myelotoxicity and dose intensity of chemotherapy: reporting practices from randomized clinical trials. J Natl Compr Canc Netw.

[3] Zhong XY, Zhang B, Asadollahi R (2010). Umbilical cord blood stem cells: what to expect. Ann NY Acad Sci.

[4] Wagner JE, Gluckman E (2010). Umbilical cord blood transplantation: the first 20 years. Semin Hematol.

[5] Sun HP, Zhang X, Chen XH (2012). Human umbilical cord blood-derived stromal cells are superior to human umbilical cord blood-derived mesenchymal stem cells in inducing myeloid lineage differentiation in vitro. Stem Cells Dev.

[6] Yang WZ, Zhang Y, Wu F (2010). Safety evaluation of allogeneic umbilical cord blood mononuclear cell therapy for degenerative conditions. J Transl Med.

[7] Zhang XZ, Xu YL, Tian R (2003). The research and application of cord blood plasma. Lin Chuang Xue Ye Xue Za Zhi.

[8] Liao C, Liu B, Huang Y (2001). Establishment of cord blood stem cell bank and its clinical application. Zhonghua Xue Ye Xue Za Zhi.

[9] Isasi R, Dalpe G, Knoppers BM (2013). Fostering public cord blood banking and research in Canada. Stem Cells Dev.

[10] Wang BH, Zhang MZ, Fu XR (2013). Pathogenesis, prevention and treatment of chemotherapy and radiotherapy induced myelosuppression: the state of the art. Zhong Liu Ji Chu Yu Lin Chuang Za Zhi.

[11] Duong CD, Loh JY (2006). Laboratory monitoring in oncology. J Oncol Pharm Pract.

[12] Lv M, Huang XJ (2012). Allogeneic hematopoietic stem cell transplantation in China: where we are and where to go. J Hematol Oncol.

[13] Riordan NH, Chan K, Marleau AM, Ichim TE (2007). Cord blood in regenerative medicine: do we need immune suppression. J Transl Med.

[14] Jiang XF, Wang GZ, Li GX (2006). Transplantation of unrelated donor umbilical cord blood in 28 patients with malignant hematopathy. Zhonghua Xue Ye Xue Za Zhi.

[15] Gu DS, Liu B, Han ZC (2006). The research and application of cord blood stem cells. Zhongguo Ke Xue Yuan Sheng Wu Ke Xue Yu Ji Shu Ju.

[16] Wang XH, Zhang XZ, Shi HZ (1999). The evaluation of Epo, G-CSF, IL-2, IL-6 in cord blood plasma. Zhonghua Xue Ye Xue Za Zhi.

[17] Biesecker LG, Emerson SG (1993). Interleukin-6 is a component of human umbilical cord serum and stimulates hematopoiesis in embryonic stem cells in vitro. Exp Hematol.

[18] Sanders M, Sorba S, Dainiak N (1993). Insulin-like growth factors stimulate erythropoiesis in serum-substituted umbilical cord blood cultures. Exp Hematol.

[19] Carow CE, Hangoc G, Broxmeyer HE (1993). Human multipotential progenitor cells (CFU-GEMM) have extensive replating capacity for secondary CFU-GEMM: an effect enhanced by cord blood plasma. Blood.

[20] An YH, Zhou RX, Zhang GJ (2000). Transplantation of cord blood hematopoietic stem cells: effects on marrow function of cancer patients on chemotherapy. Qingdao Da Xue Yi Xue Yuan Xue Bao.

